# Querying large read collections in main memory: a versatile data structure

**DOI:** 10.1186/1471-2105-12-242

**Published:** 2011-06-17

**Authors:** Nicolas Philippe, Mikaël Salson, Thierry Lecroq, Martine Léonard, Thérèse Commes, Eric Rivals

**Affiliations:** 1 LIRMM, UMR 5506, CNRS and Université de Montpellier 2, CC 477, 161 rue Ada, 34095 Montpellier, France; 2 CRBM, UMR 5237 CNRS, 1919 Route de Mende, 34293 Montpellier cedex 5, France; 3 LITIS EA 4108, Université de Rouen, 1 rue Thomas Becket, 76821 Mont-Saint-Aignan Cedex, France; 4 LIFL, UMR 8022 CNRS and Université Lille 1 and INRIA Lille-Nord-Europe, Bât. M3 - UFR IEEA, 59655 Villeneuve d'Ascq Cedex, France

## Abstract

**Background:**

High Throughput Sequencing (HTS) is now heavily exploited for genome (re-) sequencing, metagenomics, epigenomics, and transcriptomics and requires different, but computer intensive bioinformatic analyses. When a reference genome is available, mapping reads on it is the first step of this analysis. Read mapping programs owe their efficiency to the use of involved genome indexing data structures, like the Burrows-Wheeler transform. Recent solutions index both the genome, and the *k*-mers of the reads using hash-tables to further increase efficiency and accuracy. In various contexts (e.g. assembly or transcriptome analysis), read processing requires to determine the sub-collection of reads that are related to a given sequence, which is done by searching for some *k*-mers in the reads. Currently, many developments have focused on genome indexing structures for read mapping, but the question of read indexing remains broadly unexplored. However, the increase in sequence throughput urges for new algorithmic solutions to query large read collections efficiently.

**Results:**

Here, we present a solution, named *Gk *arrays, to index large collections of reads, an algorithm to build the structure, and procedures to query it. Once constructed, the index structure is kept in main memory and is repeatedly accessed to answer queries like "given a *k*-mer, get the reads containing this *k*-mer (once/at least once)". We compared our structure to other solutions that adapt uncompressed indexing structures designed for long texts and show that it processes queries fast, while requiring much less memory. Our structure can thus handle larger read collections. We provide examples where such queries are adapted to different types of read analysis (SNP detection, assembly, RNA-Seq).

**Conclusions:**

*Gk *arrays constitute a versatile data structure that enables fast and more accurate read analysis in various contexts. The *Gk *arrays provide a flexible brick to design innovative programs that mine efficiently genomics, epigenomics, metagenomics, or transcriptomics reads. The *Gk *arrays library is available under Cecill (GPL compliant) license from http://www.atgc-montpellier.fr/ngs/.

## Background

Next-generation sequencing technologies are presently being used to answer key biological questions at the scale of the entire genome and with unprecedented depth. Whether determining genetic or genomic variations, cataloging transcripts and assessing their expression levels, identifying DNA-protein interactions or chromatin modifications, surveying the species diversity in an environmental sample, all these tasks are now tackled with High Throughput Sequencing (HTS) and require different, but computer intensive bioinformatic analyses. Typically, a recent RNA sequencing experiment (RNA-Seq) produces about 8 million reads of 75 base pairs each [1], but both the yield and read length will increase [2].

Mapping the reads against a reference genome provides the genomic positions of mapped reads. For instance with RNA-Seq reads, these positions allow to know whether a gene is expressed in the studied condition. The set of mapped positions represents only part of the information needed to analyze the reads, and it can be obtained only if a genome is available. Indeed, other important information are contained in the read collection itself. For instance, to determine the frequency of haplotypes at a SNP position, one needs to align the reads related to this position. These can be obtained by considering for some length *k*, the *k*-mers overlapping the SNP and searching for the reads sharing this *k*-mer. This procedure is applicable even in the absence of a reference genome, and similar ones can be designed to search for a binding motif in ChIP-Seq reads, to determine with RNA-Seq data whether different regions of a messenger RNA sequence are susceptible to be differentially expressed, etc.

For tasks like assembly or read clustering, one needs to determine reads overlapping each other or that align partly one to another. Numerous works on similarity search algorithms have developed seed-and-extend strategies and shown that it can be performed efficiently by searching common *k*-mers between two sequences [3,4].

Surely, now and even more in the near future, we will need efficient indexing data structures to store and query large collections of reads in main memory. Up to now, a lot of computational research has been devoted to read mapping, and the most efficient tools owe their efficiency to the use of involved genome indexing data structures, like the Burrows-Wheeler transform [5]. On the other hand, the question of read indexing remains quite unexplored, although the improvements in sequencing throughput suggest that such structures will become a compulsory part of future read analysis programs. A sign supporting this view: even mapping programs now start to index both the genome and the *k*-mers of the reads to boost efficiency and accuracy [6].

Numerous works have presented data structures to index a single text, like the well known Suffix Tree (ST) or the Suffix Array (SA) [7,8]. These enable the so-called *locate *query, that is to locate all occurrences of a pattern *P *either from its sequence or from a position *j *of occurrence in the text, as well as *count *query to obtain the number of occurrences of *P*. These structures can be adapted to index a *set of texts*, where each text differ from each other; the structures are then called *generalized *Suffix Tree [9], or *generalized *Suffix Array (gSA) [10]. This is done by concatenating all texts and adding a separator symbol that does not belong to the alphabet (*e.g*., a $ for the DNA alphabet) after each text [9], or directly [10]. Then it requires to store the length of each text in an additional array to correctly answer locate queries. Such algorithms have not been adapted to *collections *of texts, where two texts may be equal in sequence but differ in their identifier. The reads obtained from sequencers form a collection, not a set.

When the total text is too large, *compressed indexes *reduce the memory needed by storing not all, but only a certain proportion of the text positions. Compression is obtained by sampling the positions to be stored, while non sampled positions need to be recomputed at run time. This enables the user to control the balance between amount of memory and query time. Hence, compression has an impact on the time needed to compute a query. Ferragina *et al*. report in a large practical evaluation of compressed text indexes, that the query time of all tested compressed indexes are between 100 and 1,000 times slower than with a plain SA for an index that is 5 times smaller [11]. The FM-index [5] is used to index all chromosomes in mapping applications [12]. However, the scalability of neither plain nor compressed indexes to collections of millions of texts has not been investigated so far. We thus address the question of indexing large collections of reads with an uncompressed index and compare its performance to a generalized suffix array and a hash table. Our structure aims to save space compared to those indexes while globally retaining queries as fast. Thus we avoid the pitfall of compressed indexes which are less space consuming but slower by orders of magnitude.

In this work, we propose a new data structure to index reads, an algorithm to build the structure, and procedures to query it. Our structure, named *Gk *arrays, is kept in main memory once built and repeatedly accessed to answer different kinds of queries like "given a *k*-mer, get the reads containing this *k*-mer (once/at least once)". One can ask both for the *k*-mer positions or simply for the reads containing it, which can prove useful in different applications. We focus on cases where millions of queries need to be computed; clearly, memory usage will be the key issue. An alternative solution is to adapt some uncompressed indexing structures designed for long texts (suffix tree or suffix array [9,13]). We compare *Gk *arrays to such an alternative and show experimentally that they process queries fast, while requiring much less memory (between 2/3 and 1/3 of a suffix array solution). We also perform experimental comparisons against a method using hash table: it shows that while the hash table method can answer quickly to queries it does not scale to large collections of reads.

If in biology the term *k*-mer is preferred, computer scientists rather use the equivalent words of *k*-factor or *k*-substring; we will stick to the term *k*-mer. The *Gk *arrays allow to answer queries related to an input *k*-mer; let us call these *k*-mer queries. Before entering the algorithms description, we list below the applications of *k*-mer queries in the analysis of High Throughput Sequencing data. The Results section will first present our data structure, its construction algorithm and the procedures to answer *k*-mer queries, then detail the experimental comparisons.

Finally, we discuss the advantages of our structure and conclude with future developments.

Note that this study does not tackle the question of read mapping, it focuses on read indexing.

### Queries and Applications

Let us give an informal presentation of the problem. We are given a collection of *q *reads of length *m *and a length of substring *k *such that *k *≤ *m*.

Suppose one is given a string *f *of length *k*; one does not know whether it appears in some of the reads or not (*i*.*e*., whether *f *is a substring of some read). In the Algorithm section, we describe a data structure in which all substrings of length *k *of the reads are ordered lexicographically. Hence, one can search for *f *using a dichotomic search in *O*(*k *log((*m *- *k *+ 1)*q*))) worst case time in this structure (the dichotomic search is the standard procedure in this context [8,9]), and determine whether at least one read contains *f *as a substring and at which position. If not, the answers to the queries below, which are all related to a sub-collection of reads containing *f*, are trivially the empty set or zero. Otherwise, one knows that *f *occurs in some read *r *of the collection at position *j*, and wishes to get some information on the other reads where *f *occurs. One wants to answer the following questions:

**Q1: **In which reads does *f *occur?

**Q2: **In how many reads does *f *occur?

**Q3: **What are the occurrence positions of *f *in the reads?

**Q4: **What is the number of occurrences of *f *in the reads?

**Q5: **In which reads does *f *occur only once?

**Q6: **In how many reads does *f *occur only once?

**Q7: **What are the occurrence positions of *f *in each read where *f *occurs only once?

**Q8: **What is the number of occurrences of *f *in the reads where it occurs only once?

We state several remarks about the queries before dwelling on applications.

1. The queries go by pairs: the first one computes a set of positions or read indices, while the second computes the cardinality of that set.

2. Note the clear semantic difference between Q1/Q2 and Q3/Q4. The answer to Q1 yields the identifiers of the reads in which *f *occurs, while that to Q3 gives **also **all its positions in the read. This clearly differs since *f *may occur several times in a read (*e*.*g*., if *f *is a poly-*A *sequence). Sometimes the positions are needed, sometimes only the reads (see below).

3. Queries Q5-Q8 are versions of Q1-Q4 constrained to a single occurrence of *f *in the reads. Of course other variants can also be computed, *e*.*g*. where the number of occurrences is limited by a user defined threshold. Since *f *is constrained to occur only once in each read, Q6 and Q8 are equivalent, and we will mention only Q6 in the sequel.

4. The data structure we propose is intended to be kept in memory and used for multiple queries.

Although this paper focuses on the data structure, its efficiency, and on the algorithms to solve these type of queries, it is important to list applications of these queries. In which context of read analysis, can one use such queries? Note that in such context, *k *is smaller than the read length. Theoretical and empirical investigations show that for instance, with *k *≥ 19 or 20, *k*-mers indicate in average a single genomic location in the human genome [14]. Such values of *k *can be computed depending on the genome length. Translated to reads or sequences: it is unlikely that two reads sharing a *k*-mer were not sequenced from the same part of the DNA. In other words, sharing a *k*-mer is a witness for having a common genomic origin.

#### Mutation detection

Putative mutations (SNP, somatic mutations, small indels) are indicated by differences between a read and a reference genome. Once the reads have been mapped to the reference genome, one analyzes the sub-collection of reads that covers a genomic position to count how many reads support the variation observed in the read or that observed in the genome. If one considers the two substrings of length *k *centered on this mutation position, one in the read and one in the genome, answering Q2 for these substrings will give an approximate count of these two haplotypes. If one needs the corresponding reads, then Q1 is the appropriate query. If only a single, or a few reads, share this *k*-mer, then a sequence error might be suspected [15].

#### Local coverage

Suppose one is given a target sequence, which can be a read or an external sequence. For each of its *k*-mer, let us call the *local coverage*, the number of reads sharing this *k*-mer (this requires a dichotomic search). The local coverage profile (*i*.*e*. a histogram of the local coverage) along the target sequence provides useful information in various contexts. For a known mRNA and an RNA-Seq experiment, the average local coverage on all *k*-mers is a proxy for the expression level of the target, while the profile enables one to distinguish the target's sub-regions expressed at different levels [16,17]. In another context, with a genomic library, taking reads as queries and looking at their local coverage profile may help to detect those overlapping the extremity of a repeated or transposable element. This may prove useful to study the distribution and evolution of these elements in the genome.

#### Clustering and assembly without a reference genome

As for Expressed Sequence Tags, it is suitable to cluster and assemble RNA-Seq reads to compute the various transcripts expressed in the assayed library [16,17]. It is necessary to detect near exact alignment between pair of reads, and this is usually performed efficiently by filtration using seeds. In such case, very efficient and sensible seeds are exact shared *k*-mers [4]. Here, the sub-collection of reads sharing a *k*-mer with a given read, as well as the *k*-mer positions, can be obtained using query Q3. The answer to Q4 can help guiding the clustering process.

Similar needs of query occur in the assembly of genomic reads [18,19]. To know with which reads one can assemble a given read without ambiguity, one may perform query Q7 using *k*-mers at the 5' or 3' extremities of the read. The obtained occurrences together with their positions will indicate the matching reads and the relative positions of read pairs for assembly.

Our application list provides examples and is by no means exhaustive. We could also mention for instance the estimation of the target genome length in assembly context, which uses *k*-mer counting [20]. Clearly, these applications are beyond the scope of this paper. However, these paragraphs underline that the proposed data structure suits the needs of read processing in various application contexts, and will provide a unified framework for building read analysis programs.

## Results and Discussion

This section contains the main contribution: a data structure to index large read collections, the *Gk *arrays. To describe it, we first introduce the notation, formalize the queries, exhibit the index data structure, give its construction algorithm, and the procedures for answering all queries. This makes the content of the Algorithms section. Then, in the Comparison section we investigate its practical usability compared to two alternatives: one based on a generalized Suffix Array (SA) and another based on a hash table. This includes theoretical and practical comparisons.

### Algorithms

Here, we detail the algorithms to build the *Gk *arrays and to answer the queries. We start by defining more formally the queries we want to answer and introduce the necessary notation.

#### Notation and definition of the queries

Let Σ be an alphabet of size σ. Σ* denotes the set of *words*, *strings *or *sequences *over Σ and, for any integer *n*, Σ*^n ^*denotes the set of words of length *n *over Σ. For a word *x*, |*x*| denotes the *length *of *x*. Given two words *x *and *y*, we denote by *xy *the *concatenation *of *x *and *y*. For every 0 ≤ *i *≤ *j *≤ |*x*| - 1, *x*[*i*] denotes the (*i *+ 1)^th ^element of *x*, and *x*[*i*.. *j*] denotes the *substring x*[*i*]*x*[*i *+ 1] . . . *x*[*j*]. Let ≤*_L _*denotes the comparison operator for the lexicographic order on words. Lexicographic ranks start from zero and all arrays are indexed from zero. For any finite set *A*, we denote its cardinality by #*A*.

The **input **consists a list *R *= (*r*_0_, . . ., *r*_*q*-1_) of *q *short sequences of length *m*, called *reads*, which are not necessarily distinct. We know that *m*, *k*, *q *∈ ℕ satisfy *m *≥ *k *> 0.

A *k*-long substring of a word is called a *k-mer*. For any *u *∈ Σ*, we denote by *F_k_*(*u*) the set of *k*-mers in *u*: *F_k_*(*u*) = {*v *∈ Σ*^k ^*| ∃*p *∈ [0, |*u*| - *k*] such that *v *= *u*[*p*. . *p *+ *k *- 1]}. Let *f *∈ Σ*^k ^*and let us denote the set of indexes of the reads in which *f *occurs by *Ind_k_*(*f*) = {*j *∈ [0, *q*[| *f *∈ *F_k_*(*r_j_*)}, and the set of *positioned occurrences *of *f *in all reads by *Pos_k_*(*f*) = {(*j*,*ℓ*) | *r_j_*[*ℓ*. . *ℓ *+ *k *- 1] = *f*}, where a positioned occurrence is given by the pair made of the read index in *R *and the beginning position of *f *in this read. Let us denote the restriction of *Ind_k_*(*f*) (resp. *Pos_k_*(*f*)) to subset of read indexes where *f *occurs only once by *UInd_k_*(*f*) (resp. *UPos_k_*(*f*)). Formally, *UPos_k_*(*f*) = {(*j*, *ℓ*) | *r_j_*[*ℓ*. . *ℓ *+ *k *- 1] = *f *and ∀*i *≠ *ℓ*, *r_j_*[*i*. . *i *+ *k *- 1] ≠ *f*}, and *UInd_k_*(*f*) = {*j *| (*j*, *ℓ*) ∈ *UPos_k_*(*f*)}. Let *i *∈ [0, *q*[, *j″ *∈ [0, *m *- *k *+ 1[, and let *f *be the *k*-mer starting at *j″ *in read *r_i_*. Note that here we require the knowledge of the pair (*i*, *j″*), which defines the *k*-mer *f*. Now, the seven *k*-mer queries can be formally defined as computing

Clearly, it appears (see Additional File 1: Proof and queries' algorithms) that the algorithms to compute *UInd_k_*(*f*), resp. *UPos_k_*(*f*), for answering Q5/Q7, simply filter *Ind_k_*(*f*), resp. *Pos_k_*(*f*), on the fly, and are thus similar to the algorithms for Q1/Q3. For place sake, we will only detail the solutions for Q1-Q4 in the sequel.

#### The index structure

Our algorithm relies on four arrays that allow to query the *k*-mers of all reads. Hence, we define a word made of the concatenation of all reads: *C_R _*= *r*_0_*r*_1 _⋯ *r*_*q*-1_. Of course, a *k*-mer that overlaps two reads in *C_R _*is not necessarily a *k*-mer of some read. Hence, we introduce a system to renumber the positions of interest in *C_R_*. The rationale behind is to save place in the *Gk *arrays by discarding the positions of overlapping *k*-mers in *C_R_*. Let us denote by  the number of distinct *k*-mers of all reads, and for the sake of legibility we set  and  (the number of interesting positions in a read and in *C_R_*, respectively). We call:

• *P*-*position*, a starting position in *C_R _*of a *k*-mer that is not overlapping two reads, *i*.*e*. an element of .

• *g*, the function that renumbers *P*-positions *in order *such that their index are consecutive; *g *is defined by:

• *Q*-*position*, an image of a *P*-position by g(.), *i*.*e*. an element of . Note that the set *Q*_pos _is not a query.

Clearly, *P*_pos _and *Q*_pos _have the same cardinality , and as (*j *≠ *j*') implies *g*(*j*) ≠ *g*(*j*'), *g *is bijective. Hence, *g*^-1 ^exists and maps a *Q*-position back to its corresponding *P*-position in *C_R_*. Proposition 1 explicits the conversion between a positioned occurrence and a *P*-position.

**Proposition 1**. *Let *(*j*, *ℓ*) *with j *∈ [0,*q*[, * be a positioned occurrence of a k*-*mer in a read*. *The corresponding P*-*position in C_R _is jm*+*ℓ*. *Conversely*, *let j*' *be a P*-*position*, *the corresponding positioned occurrence in a read is *(⌊*j*'/*m*⌋, *j*' mod *m*).

This numbering system is important for it allows us to go back and forth between a positioned occurrence in a read, its corresponding *P*-position in *C_R_*, and its *Q*-position that will be stored in our arrays.

Let *j *be a *Q*-position. We denote by *s_Q_*(*j*), resp. *f_Q_*(*j*), the suffix, resp. the *k*-mer, of *C_R _*beginning at the *P*-position *g*^-1^(*j*), *i*.*e*. *s_Q_*(*j*) = *C_R_*[*g*^-1^(*j*) . . *qm *- 1] and *f_Q_*(*j*) = *C_R_*[*g*^-1^(*j*) . . *g*^-1^(*j*) + *k *- 1]. We call *s_Q_*(*j*) a *P*-suffix. Note that all suffixes beginning at *P*-positions have different length and are pairwise distinct; thus, there are  such suffixes and they all have a different lexicographic rank. However, this may, and in real data applications will, not be the case for the *k*-mers, *i*.*e*. the *f_Q_*(*j*). We call the set {*f_Q_*(*j*) | *j *∈ *Q*_pos_} the set of *P_k_*-*factors*, whose cardinality is  with our notation.

Now, we define the *Gk *arrays:

***GkSA ***(Generalized *k *Suffix Array) is a modified Suffix Array of *C_R _*that lexicographically sorts only the *P*-suffixes,

***GkIFA ***(Generalized *k *Inverse Factor Array) is a modified Inverse Suffix Array (ISA) that stores for each *Q*-position, in position order in *C_R_*, the lexicographic rank of the *P_k_*-factors starting at the corresponding *P*-position,

***GkCFA ***(Generalized *k *Counting Factor Array) is an array that associates to a *k*-mer (actually, to its rank) its number of occurrences at *P*-positions in *C_R_*,

***GkCFPS ***(Generalized *k *Counting Factor Prefix Sum) stores the prefix sums of *GkCFA*. Since *GkCFA *and *GkCFPS *are equivalent only one of them is necessary at a time.

**Figure 1:**
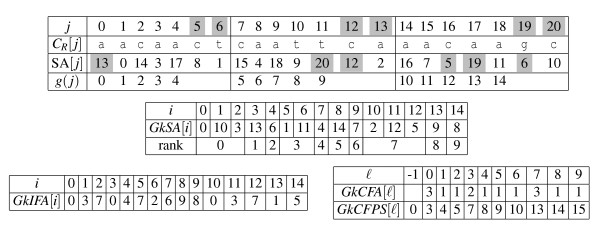
**Example of the read index data structure: the *Gk*-arrays**. Example of the read index data structure: the *Gk *arrays. Example for a collection *R *= (aacaact, caattca, aacaagc) of *q *= 3 reads of length *m *= 7 and considering 3-mers (*k *= 3). The index is composed of three tables and uses a fourth one during construction (*GkSA*, *GkIFA*, *GkCFA*, and *GkCFPS*). The first table shows the starting indices of *k*-mers in the text made by the concatenation of all reads, *C_R_*, the SA built on *C_R_*, and the function *g *that renumbers *P*-positions of *C_R _*to make them consecutive. *P*-positions are {0,1,2,3,4,7,8,9,10,11,14,15,16,17,18}; all other positions, those starting positions where the *k*-mer overlaps two reads, are displayed with a gray background (lines *j *and SA[*j*]). Line SA refers to the usual Suffix Array of *C_R_*. The *k*-mer caa occurs 4 times in *C_R _*at positions 2, 7, 12 and 16. Among those, only 2, 7, and 16 are *P*-positions. The lexicographic rank of the *P_k_*-factor starting at position 16 is given by *GkIFA*[*g*(16)] = *GkIFA*12 = 7, and the number of occurrences of the *P_k_*-factor caa is given by *GkCFA*7, which equals 3. The positions of these occurrences are thus obtained by the set {*g*^-1^(*GkSA*[*j*]) *| GkCFPS*[7 - 1] ≤ *j < GkCFPS*7} = {2,7,16}. See also Figure 2.

**Figure 2:**
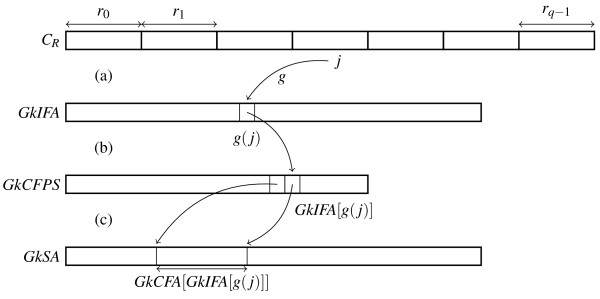
**Accessing the occurrences of a *k*-mer in the index**. Accessing the index to get the occurrences of a *k*-mer starting at position *j *in the concatenation of the reads (*i.e*., *C_R_*). Accessing *GkIFA*, *GkCFPS *and *GkSA*: (a) From *C_R _*to *GkIFA*: *g*(*j*) is the renumbered position of the *P*-position *j*. (b) From *GkIFA *to *GkCFPS*: *GkIFA*[*g*(*j*)] is the lexicographic rank of the *P_k_*-factor starting at *P*-position *j *in *C_R_*, and *GkCFPS*[*GkIFA*[*g*(*j*)]] is the number of occurrences in *C_R _*of the *P_k_*-factors of rank less than *GkIFA*[*g*(*j*)]. (c) From *GkCFPS *to *GkSA*: The positions of the occurrences of the *P_k_*-factor starting at position *j *are in *GkSA *in the range [*GkCFPS*[*GkIFA*[*g*(*j*)] -1], *GkCFPS*[*GkIFA*[*g*(*j*)]]].

Formally, the definitions are (see Figure 1 for an example and Figure 2):

• For *i *a suffix lexicographic rank and *j *a *Q*-position (*i.e. i, j *∈ *Q*_pos_),

*   GkSA*[*i*] = *j *iff *s_Q_*(*j*) has lexicographic rank *i *among the *P*-suffixes.

• For *i *a *k*-mer lexicographic rank and *j *a *Q*-position (*i.e*.  and *j *∈ *Q*_pos_),

*   GkIFA*[*j*] = *i *iff *f_Q_*(*j*) has lexicographic rank *i *among the *P_k_*-factors.

• For *i *a *k*-mer lexicographic rank (*i.e*. ),

*   GkCFA*[*i*] = #{*j *∈ *Q*_pos _*| f_Q_*(*j*) = *f_Q _*(*GkSA*[*i*])},

• For *i *a *k*-mer lexicographic rank (*i.e*. ), the definition of the prefix sum is  and 

*GkCFPS*[-1] = 0.

**Remark 1**. *The array GkCFPS is not essential to the algorithm: it is solely there to avoid multiple, time consuming computations of prefix sums over GkCFA (see GkCFPS definition above). Moreover, any value of GkCFA can also be accessed in constant time using GkCFA*[*i*] = *GkCFPS*[*i*] -*GkCFPS*[*i *-1]. *Thus, GkCFPS will be kept in memory to replace GkCFA*.

We give some useful properties of *Gk *arrays.

**Proposition 2**. *For *, *GkCFPS*[*i*] = #{*j *∈ *Q_pos _| f_Q_*(*j*) *≤_L _f_Q_*(*GkSA*[*i*])} *(Proof by induction)*.

In other words, *GkCFPS*[*i*] is the number of *P_k_*-factors having lexicographic rank less than or equal to *i*. Since *GkSA *is sorted on the lexicographic order of the *P*-suffixes, it is also sorted on the lexicographic order of the *P_k_*-factors. Hence, we get:

**Proposition 3**. *Let f *∈ Σ*^k ^such that Ind_k_*(*f*) ≠ ∅. *All occurrences of f have the same rank among the P_k_-factors, and are stored consecutively in GkSA*.

#### Construction algorithm

First, we detail the algorithm for building *GkSA*, and then the one computing *GkIFA *and *GkCFA*.

##### Computation of *GkSA*

We first build the full Suffix Array (SA) of *C_R _*using a linear time and space algorithm. Since *|C_R_| *= *mq *this first step can be done in *O*(*mq*). Then *GkSA *is obtained from SA by selecting only the *P*-positions and by renumbering them to *Q*-positions using function *g*. This second step is performed in *O*(*mq*) time and space. Moreover, *GkSA *is built in place of the Suffix Array: our algorithm allocates only the memory for the SA table. When answering Q1/Q2, each read where a given *P_k_*-factor occurs should be counted only once (even if the *P_k_*-factor occurs more than once in the read). Similarly, for Q5/Q6, we count only reads where a given *P_k_*-factor occurs exactly once. To avoid using masks on the reads, we sort in increasing order the values of *GkSA *corresponding to *P_k_*-factors sharing the same lexicographic rank (see Table in Additional File 1: Proof and queries' algorithms). The values that have to be sorted are *Q*-positions, *i.e*. integers, thus the sort can be performed in linear time on values of *GkSA *using *e.g*. radix sort [21]. The whole process takes *O*(*mq*) time and space.

##### Computation of *GkIFA *and *GkCFA*

Algorithm 1 shows how to compute jointly *GkIFA *and *GkCFA*. Its correctness proof is given in Additional File 1: Proof and queries' algorithms.

**Algorithm 1: **Computation of *GkIFA *and *GkCFA*.

**Data**: *GkSA*, *C_R_*, *k*, 

**Result**: *GkIFA *and *GkCFA*

1 begin

**2 **   *GkIFA*[*GkSA*[0]] ← 0;

**3 **   *GkCFA*[0] ← 1;

**4 **   *t *← 0;

**5    foreach **** do**

**6 **      *j *← *GkSA*[*i*];

**7 **      *j*' ← *GkSA*[*i *- 1];

**8       if ***f_Q_*(*j*) ≠ *f_Q_*(*j*') **then**

**9 **            *t *← *t *+ 1;

**10 **          *GkCFA*[*t*] ← 0;

**11 **    *GkIFA*[*j*] ← *t *;

**12 **    *GkCFA*[*t*] ← *GkCFA*[*t*] + 1;

**13   return **(*GkIFA *and *GkCFA*);

**Theorem 1**. *Algorithm 1 correctly computes the arrays GkIFA and GkCFA. (Proof in *Additional File 1*: Proof and queries' algorithms)*.

The comparison between two *P_k_*-factors (line 8) is naively performed in *O*(*k*) time, and is the only instruction of the inner loop that takes more than constant time. Hence, the computation of both *GkIFA *and *GkCFA *is performed in *O*((*m - k*)*qk*) time. Let us emphasize the simplicity of the algorithm, which explains the fast construction times obtained in practice.

**Remark 2**. *Once the values of GkCFA have been calculated, one can compute the values of GkCFPS in-place in O*((*m *- *k*)*q*) *time (see Remark 1)*.

#### Answering the queries

Assume the *Gk *arrays have been built in a preprocessing step (see section Construction algorithm); we show how to answer the first four queries, starting with Q4 and Q3. Let , and let *f *be the *k*-mer starting at *j*" in read *r_i_*. This occurrence of *f *in *C_R _*is found at *P*-position *j*':= *im *+ *j*" and the corresponding *Q*-position is *j *:= *g*(*j*').

##### Q4: Computing the cardinality of *Pos_k_*(*f*)

First, we need to find the lexicographic rank of *f *among the *P_k_*-factors, which we obtain directly by setting *t *:= *GkIFA*[*j*] (by definition of *GkIFA*). The cardinality of *Pos_k_*(*f*) is simply the number of occurrences starting at *P*-positions in *C_R_*, which is given by *GkCFA*[*t*] (by definition of *GkCFA*). By Remark 1, *GkCFA*[*t*] = *GkCFPS*[*t*] - *GkCFPS*[*t - *1].

##### Q3: Computing *Pos_k_*(*f*)

By Proposition 3, all occurrences of *f *starting at *P*-positions are stored consecutively in *GkSA*. It suffices to find the lower and upper indices, denoted by *ℓ_f _*and *u_f _*respectively. By the ordering of *GkSA *all occurrences of factors smaller than *f *in the lexicographic order are stored before its occurrences in *GkSA*. Hence, by definition of *GkCFPS *and Proposition 2, we have *u_f _*= *GkCFPS*[*t*] and *ℓ_f _*= *GkCFPS*[*t *- 1]. Since *GkSA *is indexed from 0, the starting *Q*-positions of occurrences of *f *are comprised in the range [*ℓ_f_, u_f _*] in *GkSA*. The corresponding *P*-positions are obtained using *g*^-1^(.) and are then transformed into positioned occurrences with Proposition 1. This proves Theorem 2.

**Theorem 2**. *Let f be a k-mer of a read occurring at Q-position j in C_R_. Then, its lexicographic rank among the P_k_-factors is t *:= *GkIFA*[*j*]. *If we set u_f _*:= *GkCFPS*[*t*] *and ℓ_f _*:= *GkCFPS*[*t - *1] *then*

*1. the starting P-positions of f's occurrences in C_R _are *{*g*^-1^(*GkSA*[*ℓ*]) *| ℓ *∈ [*ℓ_f_, u_f _*[},

*2*. *Pos_k_*(*f*) = {(, *g*^-1^(*GkSA*[*ℓ*]) mod *m*) *| ℓ *∈ [*ℓ_f_, u_f _*[},

*3*. #*Pos_k_*(*f*) = *u_f _*- *ℓ_f_*.

Given Theorem 2, the queries regarding *Ind_k_*(*f*) can be answered as follows:

**Q1: ***Ind_k_*(*f*): = {| ℓ ∈ [*ℓ_f_, u_f_*[},

**Q2: **by counting the elements of *Ind_k_*(*f*) while computing it.

The algorithms for Q1, Q3, and Q4 are given extensively in Algorithms 2, 3, and 4. The algorithms for all other queries are included in Additional File 1: Proof and queries' algorithms.

To answer Q7, one computes *Pos_k_*(*f*) and scans it on the fly to remove reads (or the positioned occurrences) having strictly more than one occurrence of *f*. A similar approach solves Q8, and Q5. Variants of these queries where the number of allowed occurrences is constrained by a parameter can be answered similarly.

##### Complexity

Answering Q1-Q3 or Q5-Q8 requires to scan the values in *GkSA *inside the range corresponding to the *k*-mer *f*, which can be performed in *O*(*occ_Reads(f)*) time, where *occ_Reads(f) *denotes the occurrence number of *f *in the reads. Query Q4 is computed in constant time using *GkCFPS*.

**Algorithm 2: **Q1 (*Ind_k_*(*f*))

**Data**: *f *∈ ∑*^k^*, *j *∈ *P*_pos _such that *C_R_*[*j .. j *+ *k *- 1] = *f*

**Result**: The set *Ind_k_*(*f*)

1 begin

**2 **   *Ind_k _*← empty set;

**3 **   *t *← *GkIFA*[*j*];

**4 **   *ℓ_f _*← *GkCFPS*[*t - *1];

**5 **   *u_f _*← *GkCFPS*[*t*];

**6 **   prev ← *- *1;

**7    foreach ***i *∈ [*ℓ_f_, u_f _*[  **do**

**8 **      readIndex ← ;

**9       if ***readIndex *≠ *prev ***then**

**10 **         Add readIndex to *Ind_k_*; prev ← readIndex;

**11  return **(*Ind_k_*);

**Algorithm 3: **Q3 (*Pos_k_*(*f*))

**Data**: *f *∈ ∑*^k^*, *j *∈ *P*_pos _such that *C_R_*[*j . . j *+ *k - *1] = *f*

**Result**: The set *Pos_k_*(*f*)

1 begin

**2 **   *Pos_k _*← empty set;

**3 **   *t *← *GkIFA*[*j*];

**4 **   *ℓ_f _*← *GkCFPS*[*t - *1];

**5 **   *u_f _*← *GkCFPS*[*t*];

**6    foreach ***i *∈ [*ℓ_f_, u_f _*[**  do**

**7 **      readIndex ← ;

**8 **      posInRead ← *g*^-1^(*GkSA*[*i*]) mod *m*;

**9 **      Add the pair (readIndex, posInRead) to *Pos_k_*;

**10  return **(*Pos_k_*);

**Algorithm 4: **Q4 (The cardinality of *Pos_k_*(*f*))

**Data**: *f *∈ ∑*^k^*, *j *∈ *P*_pos _such that *C_R_*[*j . . j *+ *k *- 1] = *f*

**Result**: The cardinality of *Pos_k_*(*f*)

**1 begin **//*GkCFA*[*t*] = *GkCFPS*[*t*] -*GkCFPS*[*t - *1]

**2 **   *t *← *GkIFA*[*j*];

**3    return **(*GkCFA*[*t*]);

#### Practical considerations: implementation and variable read length

The value of *k*, which determines the length of *k*-mers used for querying the collection of reads, is a parameter of our index. However, the *Gk *arrays remain flexible. If for the simplicity of the presentation we have assumed until now that all reads have the same length, the whole structure can be adapted to a collection of reads having variable length. Indeed, since some sequencing technologies produce variable-length reads (*e.g*. Roche 454^®^), this adaptation is an important issue of versatility.

##### Indexing variable-length reads

We show how our method can be slightly adapted to tackle this problem. Remind that the *Gk *arrays consider the string *C_R_*, the concatenation of all reads, and save place by discarding positions at which a *k*-mer overlaps two reads. This was done efficiently by converting any read position, or *P*-position, into a *Q*-position, and conversely, using function *g*. Up to now, this function relies on the fact that the read length is fixed. Thus, we need to modify its definition to accommodate different read lengths. For this, we use a bit vector *F*, as long as *C_R_*, to record which positions in *C_R _*are *P*-positions: *j *is a *P*-position iff *F*[*j*] = 1. We implement it as a vector having rank and select capabilities [22,23]. We define these operations as

• rank_1_(*F, i*) is the number of ones in *F*[0*..i*].

• select_1_(*F, i*) is the position of the *i*-th one in *F *(or *|F| *if there is less than *i *ones in *F*).

These operations can be performed in constant time, and *F *can be stored in a compressed form needing only *|F|H*_0_(*F*) + *o*(*|F|*) bits, where *H*_0 _is the zero-th order empirical entropy of *F*. Then computing *g*(*j*) and *g*^-1^(*j*) can be easily performed with a single rank or select query. Indeed, we have *g*(*j*) = rank_1_(*F, j*), and *g*^-1^(*j*) = select_1_(*F, j*). Finally, using little extra memory, *Gk *arrays can also handle variable-length reads.

##### Implementation

*Gk *arrays are available as a reusable C++ library under a Cecill C licence (GPL compliant). It accepts standard formats for the input read collection (FASTA, FASTQ). Depending on the number of *k*-mer positions, the user should turn on the 64 bit encoding at compilation. It allows to process data sets of more than 2^31 ^positions. Default is set to 32 bit encoding. Another compilation option can be activated to handle variable-length reads (typically Roche 454^® ^datasets), otherwise by default *Gk *arrays process fixed length reads.

The data structure construction and queries algorithms are coded in standard C and C++. To reduce memory consumption, the full SA of *C_R _*is built using libdivsufsort library https://code.google.com/p/libdivsufsort/, which was chosen for its efficiency and low memory usage (see https://code.google.com/p/libdivsufsort/wiki/SACA_Benchmarks for a benchmark of up-to-date SA construction algorithms). However, its worst case time complexity is not linear in the length of the input sequence. Also the sort of values in *GkSA *inside each range corresponding to one *P_k_*-factor is performed with the quicksort algorithm. A linear time construction of the array *GkIFA *is possible by using an LCP array (array storing the length of the Longest Common Prefixes between two consecutive suffixes in the lexicographic order). However, building this array would need at least 9*mq *bytes with Manzini's algorithm [24].

We implemented two versions of the *Gk *arrays: one which indexes only fixed-length reads, and another for variable-length reads. When not stated otherwise, *Gk *arrays refers to the implementation for fixed-length reads. For managing variable-length reads we used Sux http://sux.dsi.unimi.it/, an implementation of bit vectors with rank and select operations.

### Theoretical and experimental comparisons

The sequencing capacity of new technologies continues to improve. Managing ever increasing read collections will be a major bottleneck in the bioinformatic analysis of High Throughput Sequencing data. The *Gk *arrays implement one solution to read indexing. If plain, as well as compressed, indexing data structures have been described in the litterature (cf. Introduction), their ability to handle large read collections have not been investigated. As we seek to optimise in practice the memory consumption, the construction time, and query running time, we will compare *Gk *arrays to two other uncompressed indexes: a generalized SA (gSA) and hash tables. We choose these two alternatives for they represent different approaches to read indexing. Among the uncompressed text indexes that have been generalized to handle a set of texts, the gSA is reckoned to be one of the most memory efficient and has been preferred to hash tables or the suffix tree in other contexts [9,25]. On the other side, the optimisation of web search engines have triggered recent development of highly efficient hash tables, like Google sparse hash http://code.google.com/p/google-sparsehash or the hash tables from SGI extension of the C++ Standard Library http://www.sgi.com/tech/stl. It is thus instructive to also compare *Gk *arrays to state of the art hash tables. As explained in Introduction, compressed indexes save memory but induce much longer running times to answer queries compared to plain indexes, and have been excluded from this comparison. Nevertheless, designing efficient compressed read indexes is a challenging future research avenue, which could be addressed by compressing the *Gk *arrays.

#### A generalized Suffix Array (gSA) solution

We detail here the solution based on a generalized Suffix Array (gSA) to index a collection of reads, all reads having the same length. We call it the *gSA solution*. In fact it indexes the string made of the concatenation of all reads, *C_R_*. The preprocessing consists in building the generalized Suffix Array (gSA), the Inverse Suffix Array (ISA), and the Longest Common Prefixes (LCP) array of *C_R_*. The gSA is built using the same algorithm than for *Gk *arrays (libdivsufsort). The *ISA *is built by scanning the gSA in *mq *time, while the *LCP *array is also constructed in linear time using an efficient algorithm [26]. The tables are built in this order and add up in term of memory footprint.

**Figure 3:**
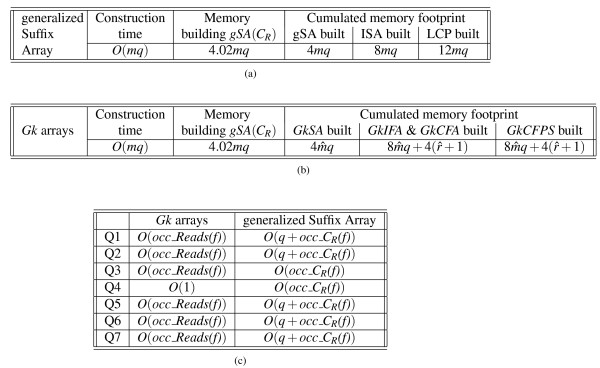
**Comparing the complexities of the *Gk *arrays and generalized Suffix Array based solutions**. Comparing the complexities of *Gk *arrays and of the generalized Suffix Array solutions. A complexity is an expression that evaluates the running time or memory usage in function of parameters describing the input size. The construction time and space complexities of the index for *q *reads of length *m *having  distinct *k*-mers are given for the generalized SA in (a), and for the *Gk *arrays in (b). We detail the cumulative space complexity during the construction of the gSA, and after the main steps of the construction algorithms. I.e.: once the gSA, the ISA, and the LCP arrays are built in (a), and once *GkSA*, *GkIFA*, and *GkCFPS *are built in (b). In (c) we give the time complexities for answering queries Q1-Q7 with a *k*-mer denoted by *f*. The procedures for the gSA depends on *occ_C_R_(f)*, the occurrence number of *f *in the text made by the concatenation of all reads (*i.e*. in *C_R_*), while those for the *Gk *arrays depends on *occ_Reads(f)*, the occurrence number of *f *in all reads, and we know that *occ_Reads(f) *≤ *occ_C_R_(f)*.

**Figure 4:**
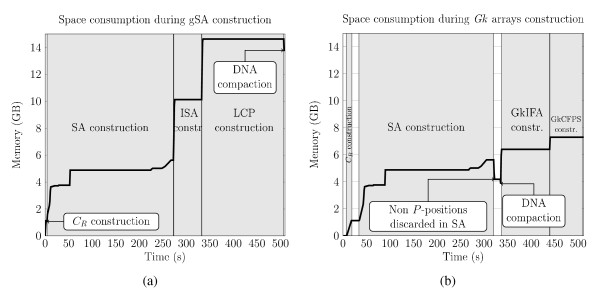
**Evolution of memory footprint during the construction of the generalized Suffix Array and of *Gk *arrays**. Evolution of memory footprint during the construction of the generalized Suffix Array (a) and of *Gk *arrays (b) when indexing 15 million 75 bp reads with *k *= 25.

In Figure 3(a) and 3(b), we compare the time and space complexities of gSA and *Gk *arrays solutions. Since both start by building *gSA*(*C_R_*) and this is the dominant term of the time complexity, we obtain *O*(*mq*) time complexity: the space occupied during the construction of that table alone is 4.02*mq*, while it amounts to 4*mq *once built [27]. The last three columns of these tables show how the cumulated memory footprint evolves after each step during construction. We also monitored the memory footprint evolution during the construction of gSA and of *Gk *arrays and illustrate these graphically in Figures 4(a) and 4(b), respectively. For the gSA the three tables add up in memory and each takes 4*mq *space. With *Gk *arrays

1. the *GkSA *table replaces *gSA*(*C_R_*) in memory and takes only ,

2. *GkIFA *takes an additional  while *GkCFA *occupies  with  denoting the number of distinct *P_k_*-factors, and

3. finally the *GkCFPS *replaces *GkCFA *and takes exactly the same space.

**Figure 5:**
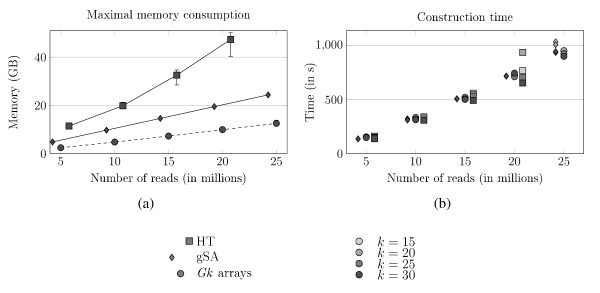
**Memory and construction time comparison between the Suffix Array solution, the hash table and the *Gk *arrays**. Memory and construction time comparison between the generalized Suffix Array solution (gSA), the hash tables (HT) and the *Gk *arrays. K562 dataset is used for that experiment, with 5 million to 25 million reads. The length of *k*-mers ranges from 15 to 30. gSA plots have been shifted left and HT plots have been shifted right for easing the reading. (a) Maximal memory usage while constructing the index and querying it. The error bars represent the space consumption depending on the value of *k*. (b) Construction time for the three indexes on the same data as for the maximal memory consumption. The levels of gray on the plots represent the value of *k*.

In total, gSA takes 12*mq *bytes of memory, while *Gk *arrays occupy  bytes (with 32-bit integers), and  is smaller than *m*. This explains why the memory footprint of *Gk *arrays remains smaller in practice than that of gSA (Figures 4(a) and 4(b)), even for varying *k *values (see Figures 5(a), 4(a) and 4(b)). Indeed, the gain of memory provided by *Gk *arrays increases with both *k *and *q*. If *k *is small, each *k*-mer tends to occur more in average, and thus , meaning that *GkCFPS *is much smaller than the LCP array. If *k *is large then 4(*m*-*k*+1)*q *≪ 4*mq *and thus, *GkSA *plus *GkIFA *tables occupy much less place than the gSA and ISA tables. This constitutes, in almost all cases, a saving of at least 12(*k *- 1)*q *bytes.

Locating a *k*-mer in the reads can be done with a binary search in *O*(*k *+ log *qm*) worst case time with gSA using SA and LCP arrays and  worst case time with *Gk *arrays using *GkSA*. (We recall that the binary search is the standard procedure in this context [8,9]).

However, Manber and Myers [8] mentioned that a simple improvement over the classical binary search (namely remembering the minimum length between the longest common prefix of the left and middle elements and the longest common prefix of the right and middle elements at each step of the binary search) permits to run in practice as fast as a  worst case method (see also [9] Section 7.14.3 page 152).

Thus, starting from a *k*-mer, rather than from a position, when answering the queries will bring an overhead similar in practice for the gSA and *Gk *arrays.

**Algorithm 5: **Q1 (*Ind_k_*(*f*)) with the generalised Suffix Array solution

**Data**: *f *∈ ∑*^k^*, *j *∈ *P*_pos _such that *C_R_*[*j .. j *+ *k - *1] = *f*

**Result**: The set *Ind_k_*(*f*)

1 begin

**2 **   *Ind_k _*← empty set;

**3 **   Initialize the whole bit vector, *D*, to zero;

**4 **   *i *← *ISA*[*j*];//starting position of* f *occurrences in SA

5    repeat

**6       if **** then**

         //the occurrence position does not overlap two reads

**7 **         readIndex ← ;

**8          if ***D*[*readIndex*] ≠ 1 **then**

            //we have not found an occurrence in this read yet

**9 **            Add readIndex to *Ind_k_*;

**10 **          *D*[readIndex] ← 1;

**11 **    *i *← *i *+ 1;

**12   until **(*i *≥ *qm*) *or *(*LCP*[*SA*[*i*], *SA*[*i *+ 1]] *< k*);

**13   return **(*Ind_k_*);

Nevertheless although we consider the same input, a position *j *of occurrence of the *k*-mer in a read, answering queries differ between the *Gk *arrays and gSA solutions. Indeed, since the gSA stores all positions in *C_R_*, we need to filter out positions of *k*-mers that overlap two reads in *C_R _*to keep only *P*-positions. This adds instructions to the procedure compared to that for the *Gk *arrays: see line 6 in Algorithm 5, which gives the algorithm for query Q1 with the gSA. For answering queries Q1 and Q2, we must perform another slight modification: we use a binary mask for dealing with duplicate *k*-mers in a same read. This mask is stored in a binary vector *B *having *q *bits, one bit per read. The bit corresponding to a read is set to one whenever the *k*-mer has been found to occur in that read, and subsequent occurrence positions in that read will be filtered out if the corresponding bit is set (lines 8 and 10 in Algorithm 5).

Assume we query on a *k*-mer *f *from one of its occurrence position *j*. Let us denote by *occ_C_R_(f) *the number of occurrences of *f *in *C_R_*, including those overlapping two reads (*i.e*., starting at non *P*-positions), and by *occ_Reads(f) *the number of its occurrences that are totally included in a read (*i.e*., those starting at *P*-positions). For Q1/Q2, Q5-Q7, we obtain with gSA a complexity of *O*(*q *+ *occ_C_R_(f)*) since one initializes the bit vector *B *of size *q *and scan all *occ_C_R_(f) *occurrences. While with *Gk *arrays, the complexity depends linearly on *occ_Reads(f) *and we know that *occ_Reads(f) *≤ *occ_C_R_(f)*.

For Q3/Q4, there is no need of a bit vector with the gSA method, hence their complexity is *O*(*occ_C_R_(f)*), for one needs to scan positions in the gSA using the ISA and the LCP arrays. However, *Gk *arrays offer a complexity of *O*(*occ_Reads(f)*) for Q3 and *O*(1) for Q4. We summarize all queries time complexities in Figure 3(c).

**Remark 3**. *To avoid scanning occ_C_R_(f) entries, an alternative solution consists in delimiting reads inside C_R _using a separator. This solution would lead to a space overhead of q bytes for lowering the time complexity to occ_Reads(f). However we did not retain this solution since our goal is to diminish the space complexity and this solution would not improve much the time complexity*.

#### A solution based on a hash table

An alternative solution is to index all *k*-mers in a hash table and to store for each read the list of its occurrence positions in the read collection. This list will contain pairs of integers: the read index in the collection, and the starting position of the *k*-mer in that read. The read index can be stored on a 32-bit integer, while a 16-bit integer suffices for the starting position. In such a case, storing the text is not necessary. The number of entries is the number of distinct *k*-mers in the read collection, *i.e*. our parameter . Generally,  is small compared to 4*^k ^*for values of *k *in [15,60]. Hence the hash table will be sparsely populated. We tried several implementation of state of the art hash tables: the Google sparse and dense hash arrays, and that from SGI extension of the C++ Standard Library (called hash map).

Preliminary experiments have shown that Google sparse requires significantly much longer to build than SGI hash map, while having a lower memory footprint. With 20 million 75 bp reads, Google sparse hash occupies one third of the memory needed by the SGI hash map, but it takes thrice more time to build. On the contrary Google dense hash tables takes twice more memory, and offers only similar construction time. Hence, SGI extension hash map exhibited the best compromise in term of memory consumption and construction time compared to Google implementations. Thus, we choose SGI extension implementation for the comparison with *Gk *arrays.

#### Experimental settings

We tested index structures on three datasets.

1. We used a collection of 40 million Illumina^® ^RNA-Seq reads of length 75 from a human K562 library taken from the RGASP data (Accession number GM12878 at http://www.gencodegenes.org/rgasp with permission from B. Wold). We call it the K562 dataset.

2. We compiled several lanes of Roche 454^® ^genomic sequencing to obtain a collection of 2.8 million reads ranging from [40,3000] bp with an average read length of 523 bp. These were sequenced on a Roche 454^® ^GS FLX platform with Titanium chemistry for the Khoisan genome project [28]. We call it the Khoisan dataset.

3. As much longer fixed length reads are not yet available, we constructed a collection of fixed length reads by slicing the Khoisan reads in non-overlapping pieces of 150 bp. We obtained 25 millions of 150 bp reads, a read length that will soon be generated on High Throughput Sequencing platforms.

In the first and third collections, reads have a fixed length, while in the second their length varies. The experiments were performed on an Intel Xeon 2.27 GHz equipped with 48 GB of main memory, and running Linux 2.6.18 with C++ compiled using gcc version 3.4.6 and -02 -funroll-loops options.

#### Experimental comparison

The use of read indexing raises three questions: how much computing resources does the index demand? Is it scalable? How fast can it answer large number of queries? Clearly the resources will depend on the number of reads (parameter *q*), their lengths and on the length of *k*-mers (parameter *k*). We compare three solutions: a hash table (HT), a generalized Suffix Array (gSA), and *Gk *arrays.

##### Scalability

We measured the construction time and amount of memory taken by all solutions for various numbers of reads and *k*-mer lengths. Figure 5(a) plots the maximal memory footprint on K562 data. At this scale, the value of *k *impacts only the hash table size; its influence on the gSA and *Gk *arrays is not visible on that graph. Second, the solutions can be ordered as follows: *Gk *arrays take the less memory, followed by the gSA, and then the hash table. This order is irrespective of the read number. For *k *= 20 *e.g*., *Gk *arrays use 10 GB, the gSA uses 20, and the HT 44, and the curves clearly indicate that these differences increase with the number of reads. Whatever the value of *q*, the hash table requires twice as much memory as the gSA, which itself takes at least 70% more memory than *Gk *arrays. With 25 million reads the hash table saturates the memory, with 30 million the gSA also does, while the *Gk *arrays constitute the only solution able to index the whole collection, 40 million reads, on that computer. Note that in both cases, the 64-bit implementation of gSA and *Gk *arrays have to be used to index that amount of reads. For the whole read collection, *Gk *arrays needs at most 43 GB (*k *= 15) and at least 36 GB (*k *= 30).

For all solutions, construction times increase linearly with the number of reads as expected (Figure 5(b)). It remains very similar between the gSA and *Gk *arrays, which both takes *e.g. <*1000 s. for 25 million reads. The influence of *k *is clearly visible on the hash table for 20 million reads: its construction time decreases with *k *because the parameter  also does (for a given number of reads). As long as they fit in memory, all compared solutions offer practical construction times.

**Figure 6:**
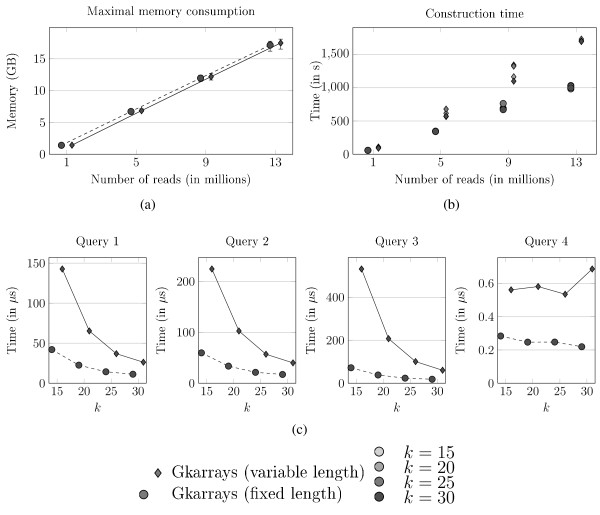
**Comparing *Gk *arrays with fixed and variable length reads**. Experiments on the sliced Khoisan dataset (150 bp-reads) with *Gk *arrays (fixed-length reads and variable-length reads). (a) Maximal space consumption of each index for several read numbers and values of *k *(the error bars represent the variation in space usage, depending on *k*). (b) Index construction time (the lighter gray corresponds to the smaller *k*). (c) Query computation time for 13,000,000 reads depending on different *k *values.

We examined the behavior of *Gk *arrays on much longer reads, 150 bp, when variable-length read option is activated and when it is not. Figure 6(a) plots space consumption, while Figure 6(b) records the construction time for both options.

We see that adding a bit vector is not space consuming since there is little difference between the two methods (Figure 6(a)). For 13 million reads, the difference is, at most, of 300 MB between the two methods. In Figure 6(b), we plotted the construction time for both indexes. The variable length read implementation becomes slower when the number of reads grows, compared to the fixed length *Gk *arrays. This shows that despite a constant-time theoretical complexity for rank and select operations; there is a dependency on the length of the bit vector in practice. However, the construction time remains reasonable in the variable case.

**Figure 7:**
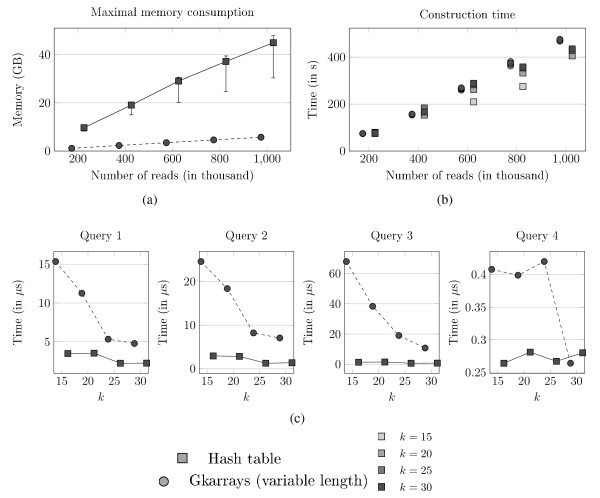
**Comparing hash table with *Gk *arrays on variable-length reads**. Experiments on the Khoisan Roche 454^® ^dataset (variable length reads) with a hash table and *Gk *arrays (with variable-length read option). (a) Maximal space consumption of each index for several read numbers and values of *k *(the error bars represent the variation in space usage, depending on *k*). The lower space consumption corresponds to a lower *k*. (b) Index construction time (the lighter gray corresponds to the smaller *k*). (c) Query computation time for 600,000 reads depending on the value of *k*.

Figures 7(a) and 7(b) plot space and time measured for the hash table and *Gk *arrays (with variable length reads option) on the Khoisan read collection. The gSA has not been implemented to handle variable length reads; note that the relative cost would have been similar to that observed with *Gk *arrays between fixed and variable read length options. Here for one million reads, variable length *Gk *arrays require 470 s. to build vs 428 s. for the hash table, but 8 times less memory (5.5 vs 46 GB). The difference increases strongly with the read number. Above one million reads, the memory footprint of the hash table exceeds the computer memory (which is 48 GB), while *Gk *arrays index the complete collection of 2.8 million reads on the same hardware with *<*15.6 GB. Hash tables appear to be more space consuming on the Khoisan dataset than on the K562 dataset. This can be explained by the nature of the data. Roche 454^® ^sequencers offer a coverage depth much lower than Illumina's. Hence the number of distinct *k*-factors in the reads is likely to be greater with the Khoisan dataset.

##### Answering queries

We measured the mean time needed to answer 100,000 random queries of Q1-Q4. Since Q5-Q7 are slight variations of Q1-Q3 we do not report on these queries.

**Figure 8:**
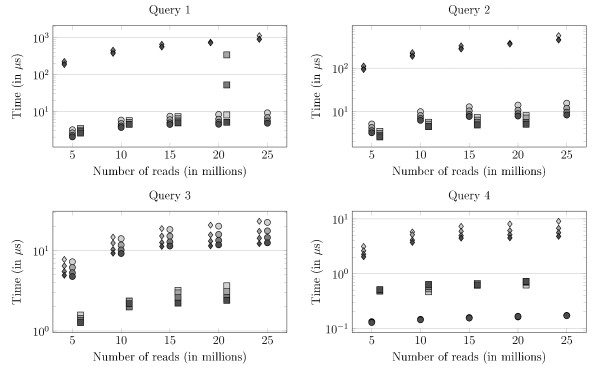
**Queries' running time comparison between the Suffix Array solution, the hash table and the *Gk *arrays**. Queries' running time comparison between the Suffix Array solution, the hash table and the *Gk *arrays. Answering the queries Q1/Q2/Q3/Q4 on K562 dataset (75 bp reads). The plots represent the average time in *μ*s over the same 100,000 queries of the corresponding type (*i.e*., Q1, Q2, Q3, or Q4). In all cases, the running time decreases when *k *increases for there are less occurrences in the reads of a say 30-mer than of a 15-mer. The *Gk *arrays are always faster than the Suffix Array; they even compute Q4 in constant time.

Figure 8 shows how the mean time for each solution vary with the number of indexed reads (*q*) and *k *on the K562 collection. Clearly, the influence of *q *is similar for all solutions, and small compared to the differences between solutions. Generally, gSA takes always longer than the hash table irrespective of the query type, and it also takes longer than *Gk *arrays for Q1-Q2 and Q4, and a similar time for Q3. The order between the hash table and *Gk *arrays depends on the query type. They are equally fast on Q1, the hash table does slightly better on Q2, clearly better on Q3, while *Gk *arrays is much faster on Q4. Anyway, for both the hash table and *Gk *arrays, the mean running time is in the order of or less than 10 microseconds for Q1-Q3, and around 0.1 microsecond for Q4 with the *Gk *arrays, meaning reasonable practical times.

For our comparison of *Gk *arrays with fixed or variable length read options, we see that the latter is becoming slower than the former (up to 7 times slower) when *k *is small, *i.e*. when the number of occurrences of *k*-mers is large. With larger *k*, the query time of the latter diminishes and becomes 2 to 3 times slower than with fixed *Gk *arrays.

With variable length reads (Figure 7(c)) the query times remain practical, but the hash table needs between 1 and 32 fold less time than *Gk *arrays depending on the query.

In summary, under various conditions *Gk *arrays are equivalent in construction time to a generalized Suffix Array or to a hash table. Compared to these solutions, they also offer reasonable query times under all circumstances; however, *Gk *arrays clearly outperform them in terms of memory footprint, the main bottleneck for processing High Throughput Sequencing data.

## Conclusions

As High Throughput Sequencing becomes widespread, computational biology will face the challenge of managing astronomical quantities of short sequences. Mining such amount of sequences is feasible if the sequences are indexed in a preprocessing step. An index is a data structure that, like a telephone book, enables one to find easily a piece of information. For some value *k*, it records the positions of all *k*-mers in the reads in an organized fashion to minimize the memory usage. Then finding the reads related to some *k*-mer takes as long as reading the *k*-mer and listing the corresponding reads, but not as long as scanning all the reads. In other words, read indexing factorizes the results of searches, which later speeds up the numerous queries made while the index is kept in memory. Our main contribution is to propose such an index: the *Gk *arrays. They are fast to build, require less space than alternative uncompressed solutions, and can thus handle larger read collections: 40 million vs 20 million reads for the hash tables with a memory limited to 48 GB. It is a key issue in practice.

While being comparable to hash tables in terms of time efficiency, only the *Gk *arrays can completely index a large read collection (like the K562 dataset) with a memory size available on nowadays computing servers. Moreover, our index remains fast for a wide range of values of parameter *k *(the length of *k*-mers). We have also shown that *Gk *arrays are both faster and smaller than an alternative generalized Suffix Array approach. Similarly, on variable-length reads like a Roche 454^® ^dataset, *Gk *arrays can handle the whole read collection using less than 16 GB while hash tables are limited to a smaller sub-collection (about 1 million reads) on a 48 GB machine.

The *Gk *arrays answer efficiently different types of queries, but they have been optimised for queries where the searched *k*-mer is extracted from an indexed read. Sometimes one wishes to know for a given *k*-mer the reads in which it occurs and its positions inside those (*e.g*. assembly), while in other contexts one only wants the number of reads sharing this *k*-mer (*e.g*. estimation of expression level). Moreover, *Gk *arrays adapt well to variable length reads. Their scalability and versatility are key advantages, which allows to envisage multiple applications as mentioned in Introduction. However, scaling up to gigantic datasets (terabytes of data), as the ones obtained in large metagenomic projects, will require compressed read indexes. The simplicity of use of our index, and its implementation as a C++ library make it a software brick that can be easily exploited in future programs or further developed by the community.

For mapping reads on a reference sequence, solutions exist that index reads with hash tables [6,29]. For the error correction problem, other works have indexed reads with classical text indexing solutions: with a generalized suffix trie [15,30], a suffix array [31], or hash tables [32]. *Gk *arrays represent a first, attractive read indexing solution; it is specialised for this question and should suit different applications. Nevertheless, one can envisage several research perspectives. Indexing approximate *k*-mers or spaced seeds will authorize more types of queries, but will certainly increase the construction time and space requirements. Designing a dynamic construction algorithm for *Gk *arrays would futher enlarge their range of applications. Another challenge is to compress *Gk *arrays by storing sampled positions and recomputing other positions at run time, as done with the Burrows Wheeler transform [5]. This would enable the user to adapt the index to its computer memory, while sacrificing some of its performance.

## List of abbreviations used

High Throughput Sequencing: HTS; RNA: ribonucleic acid; mRNA: messenger RNA; RNA-Seq: RNA sequencing; ChIP-Seq: Chromatin ImmunoPrecipitation and sequencing; SA: Suffix Array; gSA: generalized SA; LCP: Longest Common Prefix; SNP: Single Nucleotide Polymorphism; bp: base pairs; iff: if and only if.

## Competing interests

The authors declare that they have no competing interests.

## Authors' contributions

All authors have designed the algorithm and contributed to the writing of the manuscript. NP and MS have developed the code. NP, MS, TL, ER have performed the experiments. ER supervised the manuscript redaction and submission. All authors read and approved the final manuscript.

## Supplementary Material

Additional File 1 **Proof and queries' algorithms**. Click here for file
