# Supplementary material for: Querying large read collections in main memory: a versatile data structure
Source: BMC Bioinformatics. 2011 Jun 17;12:242. doi: 10.1186/1471-2105-12-242 (PMC3163563; doi:10.1186/1471-2105-12-242)
Supplement: Additional File 1 — Proof and queries' algorithms. [file 1471-2105-12-242-S1.PDF]

Additional File 1: Proof and queries algorithms to manuscript "Querying huge read sets in main memory: a versatile data structure"

Nicolas Philippe, Mikaël Salson, Thierry Lecroq,
Martine Léonard, Thérèse Commes, Eric Rivals

November 25, 2010

Additional table: elements of *GkSA* and their rank.

i	0	1	2	3	4	5	6	7	8	9	10	11	12	13	14
$GkSA[i]$	**0**	**3**	**10**	13	6	1	11	4	14	7	2	5	12	9	8
rank	0			1	2	3		4	5	6	7			8	9

Table 1: *GkSA* with values of identical rank sorted by increasing values. Compared to Figure 1, values having rank 0 are now ordered and appear as $0, 3, 10$ instead of $0, 10, 3$.

Proof of Theorem 1

Proof of Theorem 1. We want to prove that Algorithm 1 correctly computes the arrays *GkIFA* and *GkCFA*.

Initialization on lines 2 to 4. $GkSA[0]$ is the smallest P-suffix in the lexicographic order among the P-suffixes. Therefore, its lexicographic rank is initialized to 0 (line 4), which is recorded in variable t (line 2), and until now there is only one P_k-factor lexicographically ranked 0 (line 3).

Main loop on lines 5 to 12. The array *GkSA*, which stores the P-suffixes sorted in lexicographic order, is scanned from position 1 to position $\hat{q} - 1$. The rank of the previous P_k-factor is stored in t when entering the loop. Line 8 determines whether the current P_k-factor differs from the previous one. If it is so, t is incremented by since the current P_k-factor is the next one in lexicographic order. Moreover, its counter of occurrences, $GkCFA[t]$, is initialized to zero (line 10). Hence, by induction, we know that t is the lexicographic rank of the

current P_k-factor after line 10. In which case, it is recorded in entry j of *GkIFA* (line 11). Thus, *GkIFA* is correctly computed.

Moreover, each time a P_k-factor having rank t is encountered, its counter, $GkCFA[t]$, is incremented by one (line 12). Hence, at the end of the algorithm $GkCFA[t]$ correctly stores the number of occurrences of P_k-factor having rank t, as expected from its definition. This concludes the proof. □

Algorithms for queries Q2, Q5-Q7

For each of these queries the input consits in a k-mer denoted f, which is known to occur at position j in C_R.

Algorithm 1: Q2 ($\#Ind_k(f)$)

Data: $f \in \Sigma^k$, $j \in P_{\text{pos}}$ such that $C_R[j \mathinner{..} j+k-1] = f$

Result: $\#Ind_k(f)$, the cardinality of $Ind_k(f)$

```
begin
    t ⟵ GkIFA[j];
    ℓ_f ⟵ GkCFPS[t − 1];
    u_f ⟵ GkCFPS[t];
    prev ⟵ −1;
    CIndk ⟵ 0;
    foreach i ∈ [ℓ_f, u_f[ do
        readIndex ⟵ ⌊g^{−1}(GkSA[i])/m⌋;
        if readIndex ≠ prev then
            CIndk ⟵ CIndk + 1;
            prev ⟵ readIndex;
    return (CIndk);
```

Algorithm 2: Q5 ($UInd_k(f)$)

Data: $f \in \Sigma^k$, $j \in P_{\text{pos}}$ such that $C_R[j \,..\, j+k-1] = f$

Result: The set $UInd_k(f)$, subset of $Ind_k(f)$ where f occurs only once

```
begin
    UInd_k ⟵ empty set;
    t ⟵ GkIFA[j];
    ℓ_f ⟵ GkCFPS[t − 1];
    u_f ⟵ GkCFPS[t];
    prev ⟵ ⌊g^{-1}(GkSA[ℓ_f])/m⌋;
    count ⟵ 1;
    foreach i ∈ ]ℓ_f, u_f[ do
        readIndex ⟵ ⌊g^{-1}(GkSA[i])/m⌋;
        if readIndex ≠ prev then
            if count = 1 then
                Add prev to UInd_k;
            count ⟵ 1;
            prev ⟵ readIndex;
        else
            count ⟵ count + 1;
    if count = 1 then
        Add prev to UInd_k;
    return (UInd_k);
```

Algorithm 3: Q6 ($\#\mathit{UInd}_k(f)$)

Data: $f \in \Sigma^k$, $j \in P_{\text{pos}}$ such that $C_R[j \mathinner{.\,.} j+k-1] = f$

Result: $\#\mathit{UInd}_k(f)$, the cardinality of $\mathit{UInd}_k(f)$

begin
- $t \longleftarrow \mathit{GkIFA}[j]$;
- $\ell_f \longleftarrow \mathit{GkCFPS}[t-1]$;
- $u_f \longleftarrow \mathit{GkCFPS}[t]$;
- prev $\longleftarrow \lfloor g^{-1}(\mathit{GkSA}[\ell_f])/m \rfloor$;
- CUIndk $\longleftarrow 0$;
- count $\longleftarrow 1$;
- **foreach** $i \in]\ell_f, u_f[$ **do**
  - readIndex $\longleftarrow \lfloor g^{-1}(\mathit{GkSA}[i])/m \rfloor$;
  - **if** *readIndex* $\neq$ *prev* **then**
    - **if** count $= 1$ **then**
      - CUIndk $\longleftarrow$ CUIndk $+ 1$;
    - count$\longleftarrow 1$;
    - prev $\longleftarrow$ readIndex;
  - **else**
    - count $\longleftarrow$ count $+ 1$;
- **if** count $= 1$ **then**
  - CUIndk $\longleftarrow$ CUIndk $+ 1$;
- **return** (CUIndk);

Algorithm 4: Q7 ($UPos_k(f)$)

Data: $f \in \Sigma^k$, $j \in P_{\text{pos}}$ such that $C_R[j \,..\, j+k-1] = f$

Result: The set $UPos_k(f)$, subset of $Pos_k(f)$ where f occurs only once

begin
    $UPos_k \longleftarrow$ empty set;
    $t \longleftarrow GkIFA[j]$;
    $\ell_f \longleftarrow GkCFPS[t-1]$;
    $u_f \longleftarrow GkCFPS[t]$;
    prev $\longleftarrow \lfloor g^{-1}(GkSA[\ell_f])/m \rfloor$;
    posPrev $\longleftarrow g^{-1}(GkSA[\ell_f]) \mod m$;
    foreach $i \in \,]\ell_f, u_f[$ **do**
        readIndex $\longleftarrow \lfloor g^{-1}(GkSA[i])/m \rfloor$;
        posInRead $\longleftarrow g^{-1}(GkSA[i]) \mod m$;
        if $readIndex \neq prev$ **then**
            Add the pair (prev, posPrev) to $UPos_k$;
            prev $\longleftarrow$ readIndex;
            posPrev $\longleftarrow$ posInRead;
    return ($UPos_k$);
